# Binge-Like Sucrose Self-Administration Experience Inhibits Cocaine and Sucrose Seeking Behavior in Offspring

**DOI:** 10.3389/fnbeh.2017.00184

**Published:** 2017-09-27

**Authors:** Qiumin Le, Yanqing Li, Weiqing Hou, Biao Yan, Xiangchen Yu, Haikun Song, Feifei Wang, Lan Ma

**Affiliations:** State Key Laboratory of Medical Neurobiology, School of Basic Medical Sciences and Institutes of Brain Science, Fudan University, Shanghai, China

**Keywords:** paternal inheritance, intergenerational transmission, sucrose reward, rat self-administration, cocaine resistance

## Abstract

Recent studies show that emotional and environmental stimuli promote epigenetic inheritance and influence behavioral development in the subsequent generations. Caloric mal- and under-nutrition has been shown to cause metabolic disturbances in the subsequent generation, but the incentive properties of paternal binge-like eating in offspring is still unknown. Here we show that paternal sucrose self-administration experience could induce inter-generational decrease in both sucrose and cocaine-seeking behavior, and sucrose responding in F1 rats, but not F2, correlated with the performance of F0 rats in sucrose self-administration. Higher anxiety level and decreased cocaine sensitivity were observed in Sucrose F1 compared with Control F1, possibly contributing to the desensitization phenotype in cocaine and sucrose self-administration. Our study revealed that paternal binge-like sucrose consumption causes decrease in reward seeking and induces anxiety-like behavior in the F1 offspring.

## Introduction

In recent years, accumulating evidence indicates that ancestral environmental experience may result in the transmission of developmentally induced and stochastically generated phenotypes from one generation of individuals to the next, promoting non-Mendelian epigenetic inheritance and influence the development and behavior for one or a few subsequent generations (Jablonka and Raz, 2009; Miska and Ferguson-Smith, 2016). Recent findings suggest that emotional and environmental stimuli, including changes in nutritional and emotional status, or large consumption of chemicals (Roberts et al., 2007; Dias and Ressler, 2014; Bohacek and Mansuy, 2015; Toth, 2015), could facilitate behavioral plasticity and alter neurodevelopment in offspring, to adapt to the dynamic changes in the surrounding environment (Richards, 2006; Skinner et al., 2011; Manikkam et al., 2013). For example, early life stress (Franklin et al., 2010; Gapp et al., 2014a,b), fear memory (Dias and Ressler, 2014) and drug abuse (Keller et al., 1996; Crozatier et al., 2003; Bellone et al., 2011; Le et al., 2017) elicited behavioral alterations in the subsequent generations have been reported.

Appropriate responses to natural rewards were evolutionarily important for survival, reproduction, and fitness (Kelley and Berridge, 2002). There are studies delineating the nutritional aspect of food and potential impact in the offspring. *In utero* caloric undernutrition was reported to induce intergenerational transmission of glucose intolerance and obesity (Jimenez-Chillaron et al., 2009). Furthermore, paternal chronic high-fat diet ingestion lead to early onset of impaired insulin secretion and glucose tolerance in their female offspring (Ng et al., 2010). Metabolic diseases such as diabetes has been shown to exhibit paternal transmission (Wei et al., 2014). However, the incentive motivational properties of food reward on offspring has been largely neglected.

The mesolimbic reward pathway serves as critical innate driving force for pursuit of substances beneficial for survival, such as food with high content of salt, sugar and fat. The system has been extensively studied in cases of substance of abuse, that activate the mesolimbic reward pathway by directly or indirectly stimulating the firing of dopamine neurons, and causes adaptive neuronal plasticity in favor of incentive sensitization of rewarding substances (Hadad and Knackstedt, 2014; Wise and Koob, 2014; Bassareo et al., 2015). Evidence show that such effect could cause adaptions in the offspring. It was reported that parental drug exposure produces behavioral, biochemical, and neuroanatomical changes in future generations (Yohn et al., 2015). For example, maternal cocaine exposure before pregnancy can serve to enhance psychomotor sensitivity to cocaine in offspring (Sasaki et al., 2014), while paternal cocaine use causes intergenerational cocaine-resistant phenotype (Vassoler et al., 2013), anxiety (White et al., 2015), depression-like behavior (Killinger et al., 2012), impaired attention and working memory (He et al., 2006). In a recent study, we show that paternal motivation in cocaine-seeking behavior could be transmitted to the offspring and lasts for at least two generations (Le et al., 2017). And it is interesting to verify if the motivational pursuit for all rewarding events could affect their offspring, both in addictive drugs and in natural rewarding substances.

Food- and drug-induced rewarding effects, if coupled with conditional cues, could elicit incentive stimuli much stronger than the reward itself and provoke addiction-like behavior (Berridge and Robinson, 2016). In animal model of self-administration, the lever pressing activity is coupled with reinforcer supplementation and conditional stimuli. Under programs that require escalating responding to earn each reward, the motivational properties of the reinforcer could be elicited and measured (Richardson and Roberts, 1996; Roberts et al., 2007). Sucrose is a natural energy source and reward substance and provides higher reward valence even than cocaine (Lenoir et al., 2007). We thus set out to test possible effects of sucrose self-administration on the offspring.

## Materials and Methods

### Animals and Housing

Naïve Sprague-Dawley rats (F0 male and all females) were purchased from Shanghai Laboratory Animal Center, Chinese Academy of Sciences. F1 and F2 generation of the rats were bred in our own laboratory. Both F0 and their descendants were housed at 23°C on a 12-h reverse dark/light cycle (on 20:00, off 8:00), and room humidity was controlled at 40% ± 10%. Rats were housed in groups (3–4 rats per cage) and allowed free access to food and water unless otherwise specified. Animals used for behavioral tests were male rats 8–12 weeks of age, and were food-restricted and maintained at 85% original body weight. All animal treatments were in strict accordance with the National Institutes of Health Guide for the Care and Use of Laboratory Animals and approved by Animal Care and Use Committee of Shanghai Medical College of Fudan University.

### Sucrose Self-Administration and Scoring

Male rats were randomly assigned to Control group (*n* = 12) or Sucrose group (*n* = 49). Sucrose group rats were trained to press the active lever for 45 mg sucrose pellets (Bio-Serv, Flemington, NJ, USA), while those assigned to the Control group received cues without reward. The training underwent fixed ratio1 (FR1, 1 lever press/pellet) for 5 days, FR5 (5 lever presses/pellet) for 2 days, and then switched to progressive-ratio (PR) schedule, in which lever press required for each successive pellet increases by progressive increments. The PR session stopped when the rat takes more than 1 h to achieve the response requirement. Lever presses needed for the last pellet in the PR schedule was defined as the break point. Rats that achieved 50 lever presses were considered to have acquired self-administration behavior, and those that failed in the 5th FR1 session were excluded. Lever presses during FR5 and break point of individual rats were scored according to the equation (X_i_ − $\bar{\text{X}}$)/s.d. (Student’s *t*-statistic) and named as intake and motivation score, respectively. In the equation, X_i_ is the behavior value for each rat (average of lever pressed in the FR5 sessions for intake score, and break point for motivation score), $\bar{\text{X}}$ and s.d. are the mean and standard deviation of the population behavior readout (Le et al., 2017). F0 Sucrose group rats (*n* = 10) were from each of tenth percentiles of performance score (addition of intake score and motivation score), and Control F0 (*n* = 6) rats were randomly chosen from Control group. Twenty-four hours after the last session, each rat from Sucrose or Control F0 group was housed with two naïve female rats to generate F1 offspring. Six of 10 Sucrose F0 and four of six Control F0 gave birth to pups within 20–25 days after mating, and the F1 rats were housed in groups after weaning period and used for subsequent behavioral tests or mating (4 and 6 litters from Control and Sucrose F0, respectively). F2 rats were sired by crossing naïve F1 from each litter with two naïve female rats (4 and 6 litters from Control and Sucrose F1, respectively).

### Food Training and Surgery

Apart from those animals used for breeding experiment, some of the other Sucrose group rats were subjected to surgery. In F1 generation, to facilitate the rats to self-administer cocaine, rats were trained to press the active lever to get 45 mg food pellets (Bio-Serv, Flemington, NJ, USA) in the operant chambers (Med-Associates, St. Albans City, VT, USA) for 7 days. Those met the criteria of obtaining 100 food pellets per FR5 session were subjected to surgery. A silastic catheter was positioned about 3 cm into the right jugular vein, and the other end was attached to a stainless steel pedestal mounted to the rat’s skull by dental cement. The rats were allowed 7 days to recover from surgery. Catheters were flushed daily with 0.1 ml saline containing heparin (30 IU/ml) and gentamicin (0.5 mg/ml).

### Intravenous Cocaine Self-Administration

After recovery, rats were allowed to self-administer cocaine in daily sessions. When the rat pressed the active lever, an injection of cocaine (Qinghai Pharmaceutical Firm) at 0.75 mg/kg/injection over 4 s was delivered and accompanied by a conditioned cue, including the illumination of the stimulus light and an audible tone for 20 s. Presses on the inactive lever had no programmed consequences. Rats were first trained on 4 h fixed-ratio FR1 program for 5 days, then FR3 for 2 days and FR5 for 5 days. Then 6-h PR program was used, in which response requirement for each successive injection increased by progressive increments. The catheter patency was verified after PR schedule by anesthesia with chloral hydrate and data of rats with a catheter problem was excluded. Cocaine performance score for each individual was calculated the same as sucrose performance score.

### Locomotor and Cocaine Sensitivity Test

Locomotor activity was measured using commercial open field activity chambers for rats (43.2 cm × 43.2 cm × 30.5 cm, Med-Associates, St. Albans City, VT, USA). Each rat was allowed to freely explore in the chamber for 30 min. Data collection and analysis were done using Med Associates Activity Monitor program. The center area was defined as 30.5 cm × 30.5 cm, and distance traveled, time spent in the center, as were as entries to the center were recorded. In cocaine sensitivity test, rats were habituated to the open field activity chamber for 1 h, and were then administered hourly with saline or cocaine (0.25 mg/kg, i.p.). Locomotor activity was measured during each hour of the testing period.

### Elevated Plus Maze Test

Rats were subjected to habituation for 30 min in the laboratory, and then placed in the center of the elevated plus maze (Med Associates, St. Albans City, VT, USA; 70 cm above the floor, arms 50 cm × 10 cm × 40 cm) facing the closed arm and allowed to freely explore the maze for 5 min. Time spent in the open arms, and entries to the open arms during the test were recorded.

### Statistical Analysis

Active lever presses in cocaine self-administration, sucrose self-administration during FR program was analyzed with mixed linear model with repeated measurements (MMRM). Inactive lever presses and cocaine dose-induced locomotion activity was analyzed using two-way repeated-measures analysis of variance (ANOVA) followed by Bonferroni *post hoc* tests. Break point was analyzed by Mann-Whitney rank sum test. The open field test and plus maze data were analyzed with Student’s *t* test. Sample size estimation was conducted on alpha value of 0.05 and desired power of 0.80. Equal variance estimation and sample distribution were examined.* P* < 0.05 was considered statistically significant. Data are presented as mean ± SEM.

## Results

### Scoring Strategies for Evaluation of the Effect of Sucrose-Induced Reinforcement

**Figure 1:**
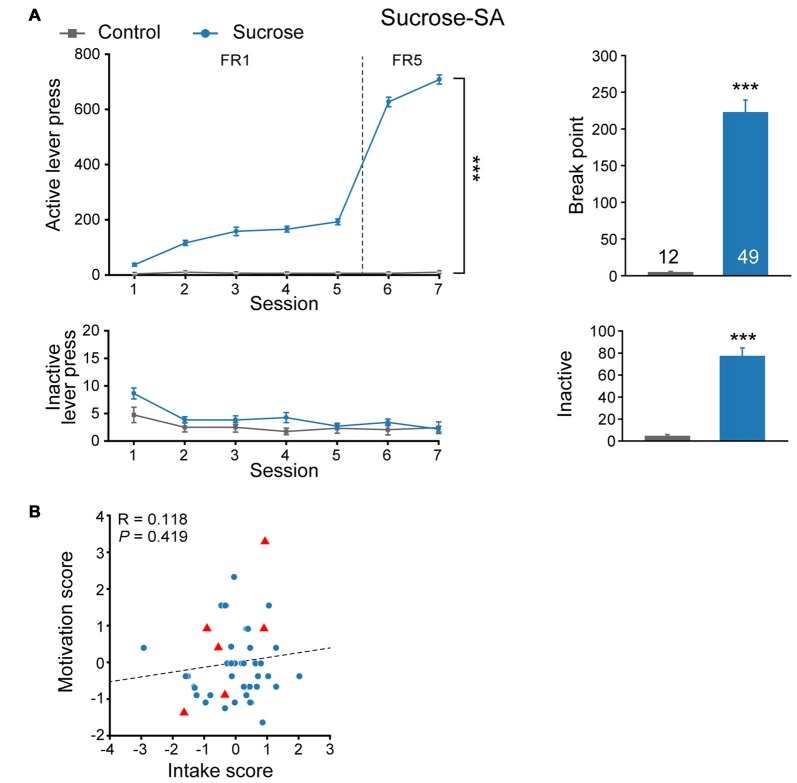
Experimental design to evaluate the performance of sucrose-seeking behavior. **(A)** Performance of sucrose self-administration behavior. Control, *n* = 12; Sucrose, *n* = 49. Control vs. Sucrose, ***P < 0.001. Data are expressed as mean ± SEM. **(B)** Intake and motivation score of each Sucrose-SA rat. Sucrose, *n* = 49. Red triangles, Sucrose F0 that gave birth to F1 pups (*n* = 6).

We first evaluated sucrose seeking behavior in a cohort of naïve SD rats. Rats were randomly assigned to sucrose or control group and subjected to an eight-session self-administration test (Figure 1A). Compared with Control group that received no reward during the training process, rats from Sucrose group exhibited significantly higher active lever presses in FR sessions (MMRM, group × FR, $\chi_{\left(1\right)}^{2}$ = 508.77, *P* < 0.001; Group, *P* < 0.001; FR, *P* < 0.001), while there was no difference in inactive lever presses (Two-way RM ANOVA, *F*_group × session_ (6,354) = 0.980, *P* = 0.439). Furthermore, rats from sucrose group were motivated to seek sucrose reward under PR test (Mann-Whitney rank sum test, *U* = 0, *P* < 0.001), and displayed higher inactive lever presses compared with Control animals (Mann-Whitney rank sum test, *U* = 34.5, *P* < 0.001), indicating compulsive binge-like sucrose reward seeking behavior. Although sucrose is a rewarding substance, we observed that rats exhibited variable lever pressing for sucrose during FR5 sessions (197–990, median 685) and break point (32–603, median 219). Next, we evaluated intake score, derived from normalized FR5 lever presses, and motivation score, from normalized PR break point for each rat individually. As there was no correlation between intake and motivation score of the cohort (Figure 1B, linear regression, *R* = 0.118, *P* = 0.419), they were considered as two distinct properties of sucrose seeking behavior, and thus the addition of intake and motivation scores were used as sucrose performance score.

### Sucrose Responding Positively Correlates with Subsequent Cocaine-Seeking Behavior

**Figure 2:**
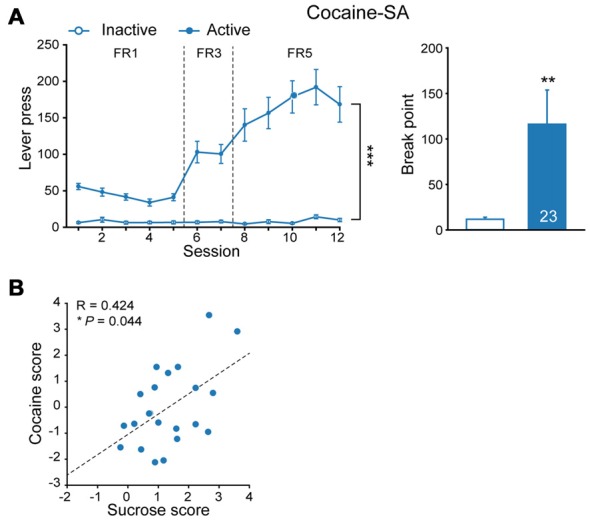
Cocaine seeking behavior correlated to prior sucrose self-administration performance. **(A)** Performance of cocaine self-administration. Sucrose, *n* = 23. Inactive vs. Active, fixed ratio (FR), ***P < 0.001, progressive-ratio (PR), **P = 0.007. Data are expressed as mean ± SEM. **(B)** Correlation of additive score of each rat in sucrose and cocaine self-administration. Sucrose, *n* = 23. *P = 0.044. Data are expressed as mean ± SEM.

Epidemiology data indicated high comorbidity rates between binge eating disorder and substance use disorders (Schreiber et al., 2013). To explore if incentive salience to sucrose is reinforcer-specific, some of sucrose-experienced rats were subjected to cocaine SA test subsequently (Figure 2A, FR, MMRM, $\chi_{\left(2\right)}^{2}$ = 170.86, *P* < 0.001; break point, Mann-Whitney rank sum test, *U* = 141.5, *P* = 0.007). Analysis of additive scores for each individual rat indicated that the number of lever press for cocaine SA was positively correlated with that of sucrose SA (Figure 2B, linear regression, *R* = 0.424, *P* = 0.044), suggesting that sucrose-seeking behavior may predict individual’s performance in response to cocaine-induced reinforcement.

### Paternal Sucrose Experience Decreases Reward Responding in F1 Offspring

**Figure 3:**
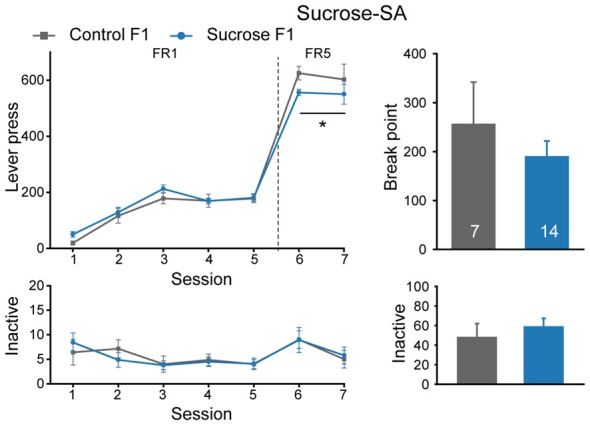
Sucrose F1 rats display slightly decreased sucrose consumption than Control F1 in FR5 sessions. Performance of Control and Sucrose F1 in FR and PR sessions. Control F1, *n* = 7; Sucrose F1, *n* = 14. Control F1 vs. Sucrose F1 in FR5, *P = 0.031. Data are expressed as mean ± SEM.

In parallel with the above experiment, some other sucrose-SA experienced male rats were bred with naïve females to see if there is an inter- or trans-generational effect, and Control rats were also bred. Six litters of Sucrose F1 and four litters of Control F1 were generated, and one naïve male from each F1 litter was used to generate F2 offspring. As shown in Figure 3, Sucrose F1 exhibited no difference from Control F1 rats in number of sessions required to achieve >50 lever presses per session in FR1 schedule (Figure 3, Mann-Whitney rank sum test, *U* = 34, *P* = 0.231), indicating comparable reward learning of these two cohorts. However, in FR5 sessions, Sucrose F1 exhibited slightly lower lever presses than the control rats (Figure 3, MMRM, group × FR, $\chi_{\left(1\right)}^{2}$ = 6.00, *P* = 0.0143; group, *P* = 0.224; FR1, *P* = 0.413; FR5, *P* = 0.031). There was no significant difference between the two groups in sucrose responding at PR schedule (break point, Mann-Whitney rank sum test, *U* = 48.5, *P* = 1.000) or inactive lever presses (FR, two-way RM ANOVA, *F*_group × session_ (6,114) = 0.280, *P* = 0.945; PR, Mann-Whitney rank sum test, *U* = 32.5, *P* = 0.232).

**Figure 4:**
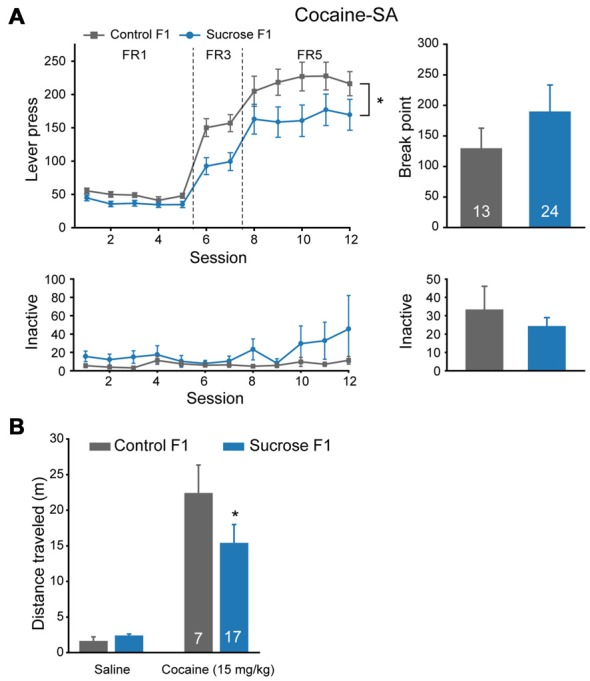
Paternal sucrose reward experience reduces cocaine self-administration responding in F1. **(A)** Sucrose F1 rats display decreased cocaine consumption than Control F1. Control F1, *n* = 13; Sucrose F1, *n* = 24. Control F1 vs. Sucrose F1, *P = 0.0285. **(B)** Sucrose F1 exhibit lower sensitivity in cocaine-induced locomotor activity. Control F1, *n* = 7; Sucrose F1, *n* = 17. Control F1 vs. Sucrose F1 at 25 mg/kg dose, *P = 0.042. Data are expressed as mean ± SEM.

As we observed significant reduced FR5 responding to sucrose in Sucrose F1 vs. Control F1, we tested the responding to cocaine to see if the effect is specific to sucrose or a general one. Significant lower lever presses were observed in Sucrose F1 as compared with Control F1 in cocaine self-administration tests (Figure 4A, MMRM, group × FR, $\chi_{\left(2\right)}^{2}$ = 20.35, *P* < 0.001; Group, *P* = 0.0285; FR1, *P* = 0.556; FR3, *P* = 0.007; FR5, *P* = 0.006), while there was no significant difference in break point (Mann-Whitney rank sum test, *U* = 48.5, *P* = 1.000) or in inactive lever presses (FR, two-way RM ANOVA, *F*_group × session_ (11,384) = 1.587, *P* = 0.100; PR, Mann-Whitney rank sum test, *U* = 245.5, *P* = 0.975). As there was no significant difference in break point between these two groups, we then asked if sensitivity to cocaine was different. We recorded locomotor responses to ascending doses of cocaine injections in a new batch of Sucrose and Control F1 rats, and found that lower locomotion response to cocaine was observed in Sucrose F1 (Figure 4B, two-way RM ANOVA, *F*_group × dose_ (1,22) = 1.770, *P* = 0.197; Control F1 vs. Sucrose F1, 25 mg/kg, *P* = 0.042), indicating desensitization to the drug.

### Paternal Sucrose Experience Increases Anxiety Level in F1 Offspring

**Figure 5:**
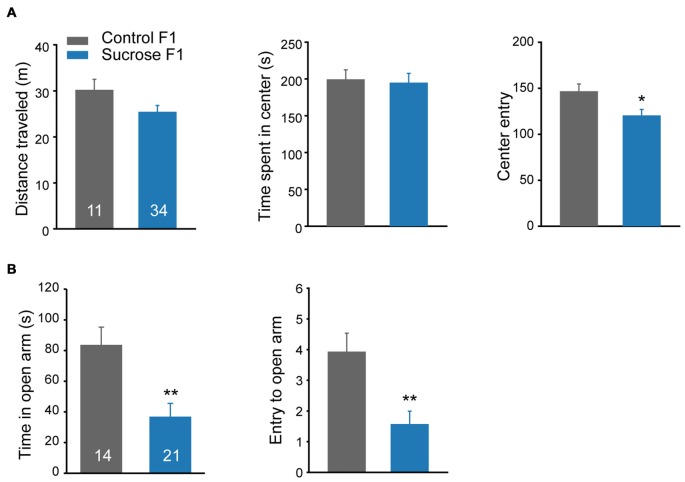
Paternal sucrose reward experience induces higher anxiety level in F1. **(A)** Sucrose F1 exhibits reduced center entry in locomotion test as compared with Control F1. Control F1, *n* = 11; Sucrose F1, *n* = 34. Control F1 vs. Sucrose F1, *P = 0.041. **(B)** Sucrose F1 rats spend less time in the open arm in the elevated plus maze test compared with Control F1 rats. Control F1, *n* = 14; Sucrose F1, *n* = 21. Control F1 vs. Sucrose F1, Time in the open arm, **P = 0.0025: entries to the open arm, **P = 0.003. Data are expressed as mean ± SEM.

Besides cocaine sensitivity, altered mobility and anxiety level may also affect lever presses in self-administration. A new batch of F1 littermates were subjected to open field activity and elevated plus maze tests. In the open field test, no difference in basal distance traveled, or time spent in the center was observed between Sucrose and Control F1 rats (Figure 5A Student’s *t*-test, total distance, *t*_(43)_ = 1.706, *P* = 0.095; time in the center, *t*_(43)_ = 0.191, *P* = 0.849). However, compared with Control F1, Sucrose F1 showed fewer entries to the center zone (Figure 5A right, Student’s *t*-test, *t*_(43)_ = 2.109, *P* = 0.041), and in elevated plus maze model, the Sucrose F1 rats spent less time (Student’s *t*-test, *t*_(33)_ = 3.271, *P* = 0.0025) and exhibited fewer entries to the open arm (Figure 5B, Student’s *t*-test, *t*_(33)_ = 3.293, *P* = 0.0024), indicating higher anxiety level, which likely contribute to the reduction of cocaine- and sucrose-responding in F1 offspring.

### Sucrose Binge Experience Does Not Cause Significant Behavior Alterations in F2 Offspring

**Figure 6:**
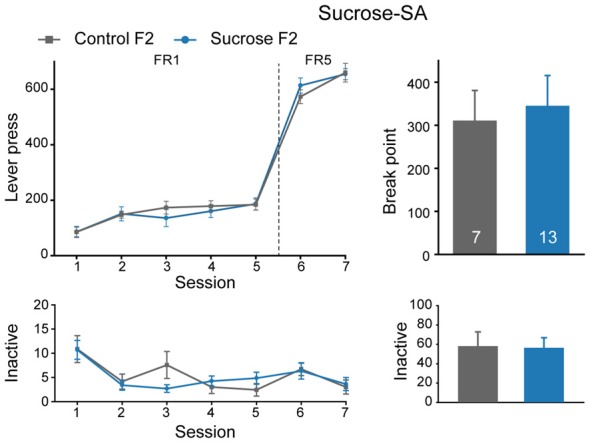
Sucrose F2 rats display comparable sucrose consumption with Control F2. Performance of Control and Sucrose F2 in FR and PR sessions. Control F2, *n* = 7; Sucrose F2, *n* = 13. Data are expressed as mean ± SEM.

We also performed sucrose self-administration experiment in F2 generation. In sucrose self-administration test, there were no significant differences in lever press learning in FR1 sessions (Figure 6, Mann-Whitney rank sum test, *U* = 37, *P* = 0.429), or sucrose seeking behavior between Sucrose and Control F2 offspring (FR, MMRM, group × FR, $\chi_{\left(1\right)}^{2}$ = 4.29, *P* = 0.0384, group, *P* = 0.380; break point, *U* = 44.5, *P* = 0.968). Taken together, sucrose binge experience could decrease both sucrose and cocaine intake in F1, but not F2 generation, indicating an inter-generational decrease in reward responding in general.

### Paternal Propensity in Sucrose Reward Seeking Behavior Are Maintained to the F1 Generation

**Figure 7:**
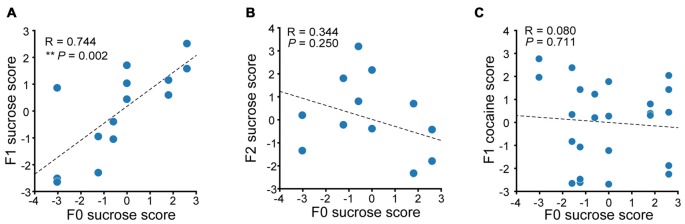
Paternal propensity in sucrose reward seeking behavior were maintained to the F1 generation. **(A)** Sucrose performance scores of F0 and F1 are positively correlated. Sucrose F1, *n* = 14. F0 sucrose score vs. F1 sucrose score, **P = 0.002. **(B)** Sucrose performance score of F0 and F2 exhibit no correlation. Sucrose F2, *n* = 13. **(C)** There is no correlation between F0 sucrose score and F1 cocaine score. Sucrose F1, *n* = 24.

We then performed correlation analysis to see if there were any relevance in the reward seeking behavior in F0 and their descendants. We first compared the sucrose performance score of Sucrose F1 rats with that of their fathers. A significant positive correlation in additive sucrose scores between F0 and F1 was observed (Figure 7A, linear regression, *R* = 0.744, *P* = 0.002). However, no significant correlation between F2 and F0 in sucrose self-administration performance was observed (Figure 7B, linear regression, *R* = 0.344, *P* = 0.250). And although there was significant positive correlation between cocaine and sucrose performance score in F0 of each tested individual, no correlation between sucrose score in F0 rats and cocaine score in F1 was observed (Figure 7C, linear regression, *R* = 0.0797, *P* = 0.711). Taken together, the maintenance of paternal incentive salience to reward was inter-generational and reinforcer-specific, as we could only observe correlated score of sucrose-SA in sucrose-experienced F0 and its F1, but not F0 sucrose score and F1 cocaine score.

## Discussion

In this study, we examined the effects of sucrose-induced reinforcement in the offspring. Despite our preliminary assumption that there should be maintenance of paternal reinforcement vigor in offspring, the observed effect is quite complicated. We recently showed that the high incentive responding to cocaine in the F0 generation could be transmitted to F1 and F2 generations (Le et al., 2017). Moreover, the inheritance of high incentive response to cocaine is contingent on high motivation, as it is elicited by voluntary cocaine administration, but not high intake of cocaine itself, which lead to resistance to cocaine in F1 generation. Combined with our previous data, we could further our hypothesis on the effect of ancestral reward experience on the subsequent generation. First of all, the maintenance of paternal reward vigor to cocaine or sucrose was reinforcer-specific, as we could only observe correlated score of sucrose-SA in sucrose-experienced F0 and its F1, but not F0 sucrose score and F1 cocaine score; and in our previous study, cocaine Addict F1 exhibited higher responding than NonAddict F1, but there was no effect in sucrose-SA experiments. Secondly, both intake of cocaine and sucrose could lead to decreased responding to cocaine in F1, reflecting a reinforcer-induced global suppression in the reward system in the subsequent generation, namely the “protective effect”, consistent with reports from Vassoler et al. (2013). Taken from these data, we speculate that, paternal reinforcing experiences could lead to reinforcer-specific memory, that lead to maintenance of paternal pursuit of the reward. In the meantime, a protective effect that suppresses the reward responding also emerged along with the maintenance. These counteracting and balancing effects may facilitate the memorization of favorable events in the offspring, and prevent individuals from excessive indulgence of the rewarding events.

However, cocaine and sucrose are naturally distinct. Cocaine is a novel (non-natural) reward, may leave epigenetic engrams in offspring to facilitate prompt and favorable adaptive responses upon their exposure (memory recall) to cocaine in subsequent generations. Sucrose, an ancestrally exposed subject, may share similar maintenance of motivation, but the phenomenon was much weaker preserved, possibly due to the fact that it is a “build-in” energy source and already settled in the reward system due to the natural selection-like mechanisms. In contrast to the results of cocaine, no correlation in sucrose score between F0 and F2 was observed. These data suggest that unlike cocaine, higher motivation for sucrose does not appear to cause transgenerational transmission. Thus it is quite feasible that natural and drug rewards may produce differential effects on offspring.

There are overlapping mechanisms underlying the neuronal circuitry of nature and drug reward. Thus it is possible that exposure to drugs can produce cross-sensitization with natural reinforcers such as sex and food, and vice versa. Indeed, studies have shown that amphetamine and alcohol exposure facilitates sugar-induced reinforcement (Cullere et al., 2014; Caprioli et al., 2015), and that restricted sucrose exposure leads to elevated motivation for cocaine (Li et al., 2016), as compared with chow-exposed animals. Epidemiological studies have also shown positive correlation of drug or alcohol craving vs. high-sugar food (Pelchat, 2002; Fortuna, 2010). In our study, sucrose responding in F0 positively correlates with subsequent cocaine-seeking behavior. However, the correlation was only observed in F0, but not transmitted to the offspring. Thus, it is likely that innate motivation for reward in general does not predispose offspring to vulnerability to addiction to an unexposed substance of abuse. Only reinforcer-specific “memory” of the father was affecting the offspring.

Heritability was traditionally thought to be dependent on the genetic material of an organism, i.e., DNA. However, there are accumulating evidence showing that non–DNA methods are also involved in transgenerational inheritance. Transgenerational epigenetic inheritance of acquired states has recently drawn widespread attention and debate (Heard and Martienssen, 2014; Nagy and Turecki, 2015; Miska and Ferguson-Smith, 2016; Bohacek and Mansuy, 2017). It is suggested that environmental stimuli may cause non-genetic germline-dependent transmission that directs offspring to promptly respond to the experience. The mechanisms underlying germline epigenetic inheritance include DNA methylation (Franklin et al., 2010; Gapp et al., 2014a; Radford et al., 2014; Skinner, 2014; Wei et al., 2014), noncoding RNAs (ncRNAs; Gapp et al., 2014a; Chen et al., 2016), histone (Vassoler et al., 2013; perhaps also protamine) post-translational modifications. In the current study, we observed non-Medelian acquisition of cocaine and sucrose resistance, as well as maintenance of paternal response vigor to sucrose in F1. Very likely, such transmission could be caused by non-genetic, i.e., “epigenetic” mechanisms. However, differential responses of naïve animals to sucrose reward might be caused by a distinct genetic or non-genetic basis. These comprehensive mechanisms interact with each other, and lead to the intriguing paternal transmission effect of sucrose binge experience. Whether it is universal principle that ancestral reward experience might exert effect in the offspring needs further evidence.

## Author Contributions

YL, WH, XY, QL, BY and HS carried out experiments, YL, XY and QL performed statistical analysis, YL, FW and LM designed the study and wrote the manuscript.

## Conflict of Interest Statement

The authors declare that the research was conducted in the absence of any commercial or financial relationships that could be construed as a potential conflict of interest.
